# Trends in SARS-CoV-2 Cycle Threshold Values in Bosnia and Herzegovina—A Retrospective Study

**DOI:** 10.3390/microorganisms12081585

**Published:** 2024-08-04

**Authors:** Almedina Moro, Adis Softić, Maja Travar, Šejla Goletić, Jasmin Omeragić, Amira Koro-Spahić, Naida Kapo, Visnja Mrdjen, Ilma Terzić, Maja Ostojic, Goran Cerkez, Teufik Goletic

**Affiliations:** 1 Clinical Center of University of Sarajevo, 71000 Sarajevo, Bosnia and Herzegovina; almamoroha@yahoo.com; 2 Veterinary Faculty, University of Sarajevo, 71000 Sarajevo, Bosnia and Herzegovina; adis.softic@vfs.unsa.ba (A.S.); sejla.goletic@vfs.unsa.ba (Š.G.); jasmin.omeragic@vfs.unsa.ba (J.O.); amira.koro@vfs.unsa.ba (A.K.-S.); naida.kapo@vfs.unsa.ba (N.K.); ilma.terzic@vfs.unsa.ba (I.T.); 3 Department of Microbiology and Immunology, University Clinical Centre of the Republic of Srpska, 78000 Banja Luka, Bosnia and Herzegovina; maja.travar@kc-bl.com (M.T.); mrdjen.visnja@gmail.com (V.M.); 4 Faculty of Medicine, University of Banja Luka, 78000 Banja Luka, Bosnia and Herzegovina; 5 Institute for Public Health FB&H, 88000 Mostar, Bosnia and Herzegovina; m.ostojic@zzjzfbih.ba; 6 Ministry of Health of the Federation of Bosnia and Herzegovina, 71000 Sarajevo, Bosnia and Herzegovina; goran.cerkez@fmz.gov.ba

**Keywords:** SARS-CoV-2, variants of concern, Ct values, decision-making tool, Bosnia and Herzegovina

## Abstract

The emergence of severe acute respiratory syndrome coronavirus 2 (SARS-CoV-2), which led to the COVID-19 pandemic, has significantly impacted global public health. The proper diagnosis of SARS-CoV-2 infection is essential for the effective control and management of the disease. This study investigated the SARS-CoV-2 infection using RT-qPCR tests from laboratories in Bosnia and Herzegovina. We performed a retrospective study of demographic data and Ct values from 170,828 RT-qPCR tests from April 2020 to April 2023, representing 9.3% of total national testing. Samples were collected from 83,413 individuals across different age groups. Of all tests, 33.4% were positive for SARS-CoV-2, with Ct values and positivity rates varying across demographics and epidemic waves. The distribution was skewed towards older age groups, although lower positivity rates were observed in younger age groups. Ct values, indicative of viral load, ranged from 12.5 to 38. Lower Ct values correlated with higher positive case numbers, while higher Ct values signaled outbreak resolution. Additionally, Ct values decreased during epidemic waves but increased with the dominance of certain variants. Ct value-distribution has changed over time, particularly after the introduction of SARS-CoV-2 variants of interest/concern. Established Ct value trends might, therefore, be used as an early indicator and additional tool for informed decisions by public health authorities in SARS-CoV-2 and future prospective pandemics. Moreover, they should not be overlooked in future epidemiological events.

## 1. Introduction

The severe acute respiratory syndrome coronavirus 2 (SARS-CoV-2) outbreak that resulted in coronavirus disease 2019 (COVID-19) has posed a serious threat to public health worldwide. SARS-CoV-2 is an RNA virus with a single-stranded, positive-sense RNA genome approximately 30 kilobases in length. The genome encodes several structural proteins including the spike (S) protein, envelope (E) protein, membrane (M) protein, and nucleocapsid (N) protein. The spike protein plays a crucial role in viral entry into host cells [1]. The proper diagnosis of SARS-CoV-2 infection is essential for the effective control and management of the disease. The detection of the presence of the virus in nasopharyngeal samples is performed by using a variety of laboratory methods and techniques, and the preferred method for diagnosing SARS-CoV-2 infection is through direct detection of the viral genome using the real-time quantitative reverse transcription-PCR (RT-qPCR) molecular technique. This technique involves converting RNA into complementary DNA (cDNA) through reverse transcription, followed by amplification of specific DNA targets using quantitative PCR [2]. In RT-qPCR, the cycle threshold (Ct) value is defined as the cycle number at which the fluorescence signal from the PCR assay exceeds a predefined threshold level, indicating the presence of the target nucleic acid [2]. However, the sampling of nasopharyngeal swabs appeared to be challenging to standardize, leading to difficulties in quantitatively analyzing the results. RT-qPCR systems primarily provide qualitative analysis, indicating the presence or absence of the virus. The cycle threshold (Ct) values obtained from RT-qPCR assays can provide a semi-quantitative estimation of the infectiousness of SARS-CoV-2-positive individuals representing the first RT-qPCR cycle at which a detectable signal appears during the assay. Some countries initially used a Ct value of >30 as a cut-off for determining the need for quarantine of SARS-CoV-2-positive patients [3]. However, subsequent studies have shown that infectious viruses can be detected even in cases where Ct values >30. Factors such as the time of sampling, quality of swabs, nucleic acid extraction method, and PCR protocol can influence the Ct value and its correlation with infectiousness [4].

Long-term epidemiological studies have provided insights into the significance of Ct values in SARS-CoV-2 PCR testing. Previous research using a cultured SARS-CoV-2 virus from nasopharyngeal specimens demonstrated that Ct values were a remarkable predictor of RNA viral load, and that the relationship between Ct values and risk of transmission was inversely related [5]. Low Ct values appear in the acute phase of infection, indicating high viral load and potentially high infectivity of positive individuals, while high Ct values usually appear in the initial phase or the final phase of infection [6]. Ct values can serve as an indicator of viral load, which is associated with the stage of infection and general infectivity. However, the direct correlation with disease severity is complex and less straightforward [7,8]. Ct values can be useful for estimating the level of infection in a population, and serve as an epidemiological early warning indicator of changes in transmission patterns. Despite their use as a marker of viral load, Ct values are not reliable for accurately assessing disease severity in individual patients due to variability in test conditions and patient factors [6,9,10]. Also, there is substantial controversy around the age groups and educational levels at which SARS-CoV-2 infection is more likely to occur [11]. However, it is essential to note that such studies are not designed to directly correlate Ct values with infectiousness or prognosis of SARS-CoV-2 infection. Instead, they help with understanding the relationship between Ct values and disease dynamics in a broader population.

Accurate laboratory methods are vital for diagnosing and managing SARS-CoV-2 infection. Although PCR-based assays provide sensitive detection, interpreting Ct values for determining infectiousness remains challenging due to the factors mentioned above [4]. There is ongoing debate about the utility of Ct values for evaluating the infectivity and severity of SARS-CoV-2 infections. While they provide insights into viral load, their effectiveness at predicting individual disease outcomes and severity remains uncertain.

However, many studies aimed at examining the association between Ct values and epidemiological trends reported a time lag in the negative cross-correlation between Ct values and new daily cases, as previously reviewed [12]. The main finding of the studies was that viral loads increased, as indicated by low Ct values, as case numbers rose toward the peak of the epidemic wave. Towards the end of epidemic waves, there was also a higher proportion of individuals with higher Ct values, indicating a decrease in viral load [12]. Large-scale data collection across multiple institutions or testing laboratories is challenged by the utilization of various PCR kits, leading to potential inconsistencies in Ct values, even when targeting identical genes. This diversity in assays may introduce noise into the aggregated Ct values, posing challenges to their interpretation. Currently, the implications of Ct values derived from different assays and gene targets for predicting epidemiological trends remain uncertain [13]. However, the global practice of using various PCR tests is unavoidable and presents a limitation in studies of this kind.

Inspired by similar study designs, we retrospectively examined data from RT-qPCR tests performed in nasopharyngeal/oropharyngeal specimens from 83,413 individuals from different geographical areas in Bosnia and Herzegovina that were conducted between April 2020 and April 2023. Samples were examined at two diagnostic facilities: the laboratory for molecular–genetic and forensic investigations of the Veterinary Faculty of the University of Sarajevo (LMGFI) and the Department of Clinical Microbiology of the University Clinical Center of the Republic of Srpska (UKC RS). The study aimed to evaluate the relationship between the Ct value and the emergence and course of the COVID-19 pandemic in Bosnia and Herzegovina, with a focus on certain age-related demographic groups.

## 2. Materials and Methods

Ct values obtained from CE-IVD certified RT-qPCR assays for the SARS-CoV-2 infection were analyzed in the ISO 17025:2018-accredited laboratories (LMGFI and UKC RS). The list of used diagnostic kits with accompanying information about the manufacturer, target genes, internal control and appropriate detection fluorophore channels is given in Supplementary Table S1.

The viral nucleic acids were extracted using either the Viral RNA Mini extraction kit (Qiagen, Hilden, Germany) or the VN143-Viral RNA extraction kit (Genolution, Seoul, Republic of Korea), with the automated nucleic acid extraction system Nextractor^®^ NX-48N (Genolution, Seoul, Republic of Korea). After preparation, 140 µL of a sample from a nasopharyngeal/oropharyngeal swab was used for the extraction process, culminating in the final elution volume of 50 µL. Five (5) µL of RNA eluate was used for each reaction, and all samples were tested in singlets. All samples were tested in singlets to accommodate the high volume of testing and to ensure the efficient use of resources. Probes targeting different genes were labelled by the appropriate fluorophores for the detection of multiple genes in one reaction. Data were acquired with the QuantStudio™ 5 Real-Time PCR System (ThermoFisher Scientific, Waltham, MA, USA) and analyzed using QuantStudio™ Design and Analysis Software v1.5.1 (ThermoFisher Scientific, USA). The presence of SARS-CoV-2 was determined by the Ct value in fluorophore-associated channels and the internal control, present throughout the complete process of extraction. A positive result, indicating the presence of SARS-CoV-2 RNA, was determined as Ct values less than 38, as recommended by the manufacturers of diagnostic kits. If the internal control had a Ct value below 35 and the appropriate fluorophore channel detecting the corresponding gene presence of SARS-CoV-2 showed a negative signal, the result was considered negative, indicating either no presence of the virus in the sample or a concentration below the detection level. If both the sample-associated channels and internal control-associated channels had no signal, the reaction and the result were interpreted as invalid.

In each reaction, three types of controls were included: negative and positive controls, as recommended by the detection kit manufacturer, and internal laboratory controls (two negative samples) that were randomly inserted on a plate before extraction. No substantial changes were made in the laboratory protocols, or in the sample collection over the analyzed period, in either diagnostic laboratory.

The research was carried out in compliance with the Helsinki Declaration and relevant institutional guidelines and ethical requirements for work with human subjects. Informed consent was obtained from all participants involved in the study. For participants under the age of 18, consent was obtained from a parent or legal guardian. The consent process was documented, and participants were informed about the study’s purpose, procedures, and benefits. Participants were assured of the confidentiality of their data. Data collection for each registered and tested individual was conducted using an epidemiological questionnaire. This questionnaire gathered personal identification details, as well as information on age, gender, COVID-19 symptoms, patient residence and, since LMGFI predominantly tested asymptomatic individuals who travelled abroad, the time and destination of the planned trip. The collected data were categorized into eight demographic groups according to age and educational level: group 1—newborns/toddlers and preschool children (0–5 years); group 2—elementary school children (6–14 years); group 3—secondary/high school children (15–19 years); group 4—youth/students (20–29 years); group 5—adults/active working population (30–49 years); group 6—adults/working population (50–64 years); group 7—retired population (65–79 years), and group 8—seniors/old population (80+ years).

Dataset cleaning, summary statistics, and visualizations were performed in Stata Statistical Software 15.1 (College Station, TX, USA: StataCorp LLC). The Kruskal–Wallis One-Way test and Dunn’s post hoc test were used to compare Ct value differences between categories. Fisher’s exact test was used to assess the difference in the aggregate prevalence of SARS-CoV-2 positive tests between categories. The Mann–Kendall test was employed to evaluate the presence of monotonic trends over time and patterns in the data. This non-parametric test was chosen for its robustness against outliers and its ability to detect trends without assuming a specific data distribution. A *p*-level of significance was set at 0.05 for all statistical tests.

## 3. Results

**Figure 1:**
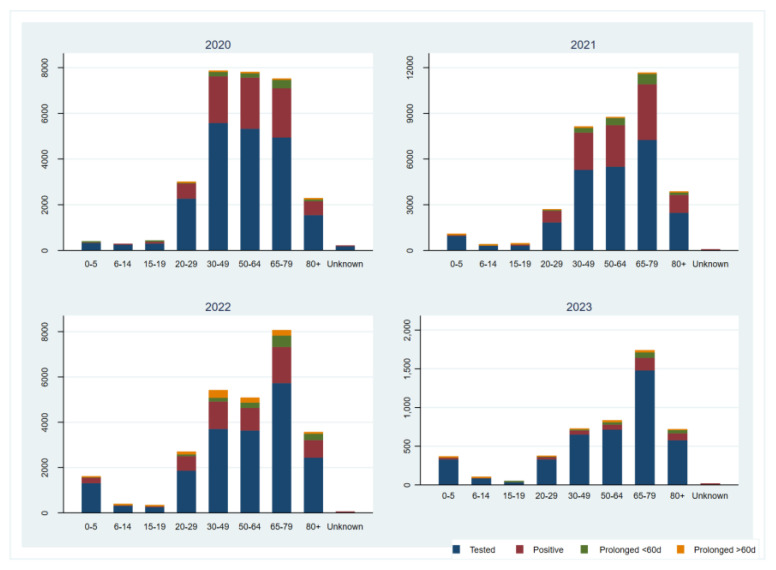
The investigated population with a number of tests performed in each age group represented per year. Prolonged positive individuals were recorded in all age groups. A total of 95.9% of individuals who tested positive had only one positive test result, 4.9% of individuals had between two and four consecutive positive test results (prolonged positive for less than 60 days), and 1.7% of individuals had between five and eight consecutive positive test results (prolonged positive for more than 60 days).

**Figure 2:**
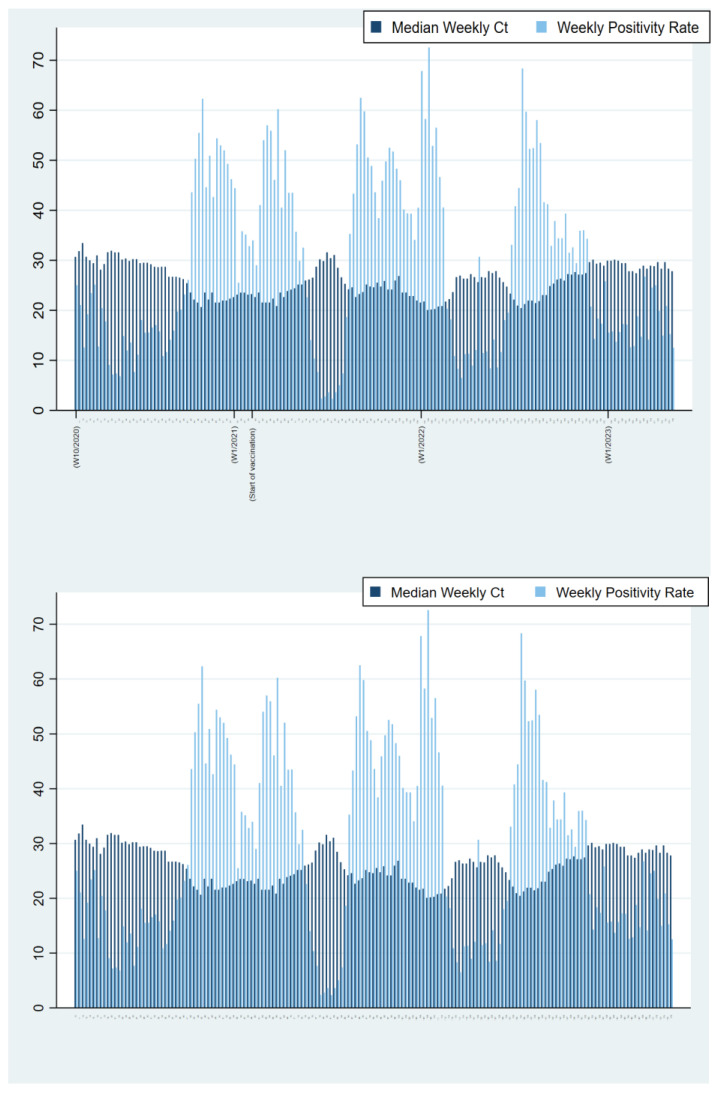
The association of weekly median Ct values and the weekly positivity rates (period April 2020–April 2023). The observed trend in the slight but constant decrease in weekly median Ct values several weeks before the exponential growth of positive cases indicates the next epidemic wave. The shown increment size is every ten weeks covered by the study, starting with the first and ending with the last week. The number of the week and the corresponding year to which the displayed data refer are given in parentheses.

In total, 170,828 tests were performed in nasopharyngeal specimens from 83,413 individuals, which represents approximately 9.3% of all SARS-CoV-2 testing in Bosnia and Herzegovina from April 2020 to April 2023 [14,15]. The samples were received from 90 out of 143 (62.9%) municipalities in Bosnia and Herzegovina. The investigated population in this study is shown in Figure 1, and a number of tests were performed for each age group, respectively. Out of all tests performed and individuals tested, 57,086 tests (33.4%) and 31,543 individuals (37.8%) were positive for the SARS-CoV-2 infection. The number of PCR tests performed varied during the epidemic, and peaked during the first, second, and third waves when more than 1400 tests per week were recorded. A total of 87.9% of individuals were tested only once, while 12.1% of the individuals underwent multiple tests (2–8 times) (Figure 1). The distribution of tests performed by age group was not uniform, with a lower number of tests in newborns/toddlers and preschool children (0–5 years), elementary school children (6–14 years), and secondary/high school children (15–19 years) compared to older age groups (Figure 1). This disproportion was evident in all epidemic years/waves. The distribution of all tests performed, and corresponding positivity rates, correlated with both the tested population structure and the diagnostic purpose of the testing (whether for request or hospitalization needs). Although testing rates varied among children, positivity rates consistently remained the lowest in these age groups during the epidemics. The trend in testing rates changed slightly during waves at the end of 2022 and 2023 with the Delta and Omicron variants (Figure 1), when an increase in the number of tested newborns/toddlers and preschool children was recorded. However, the positivity rates remained the lowest in comparison with all the other investigated age groups. No significant differences in distributions of all tests, positive ones and corresponding test positivity rates between genders, were observed in the specified age groups. Similar to the total number of performed PCR tests, the number of positive cases also fluctuated over different epidemic waves when it exceeded 1200 positive tests per week. The positivity rate substantially varied throughout the epidemic, with a weekly average of 29.5% and peaking at 72.6% in the third week of 2021 (Figure 2).

### Ct Values

**Figure 3:**
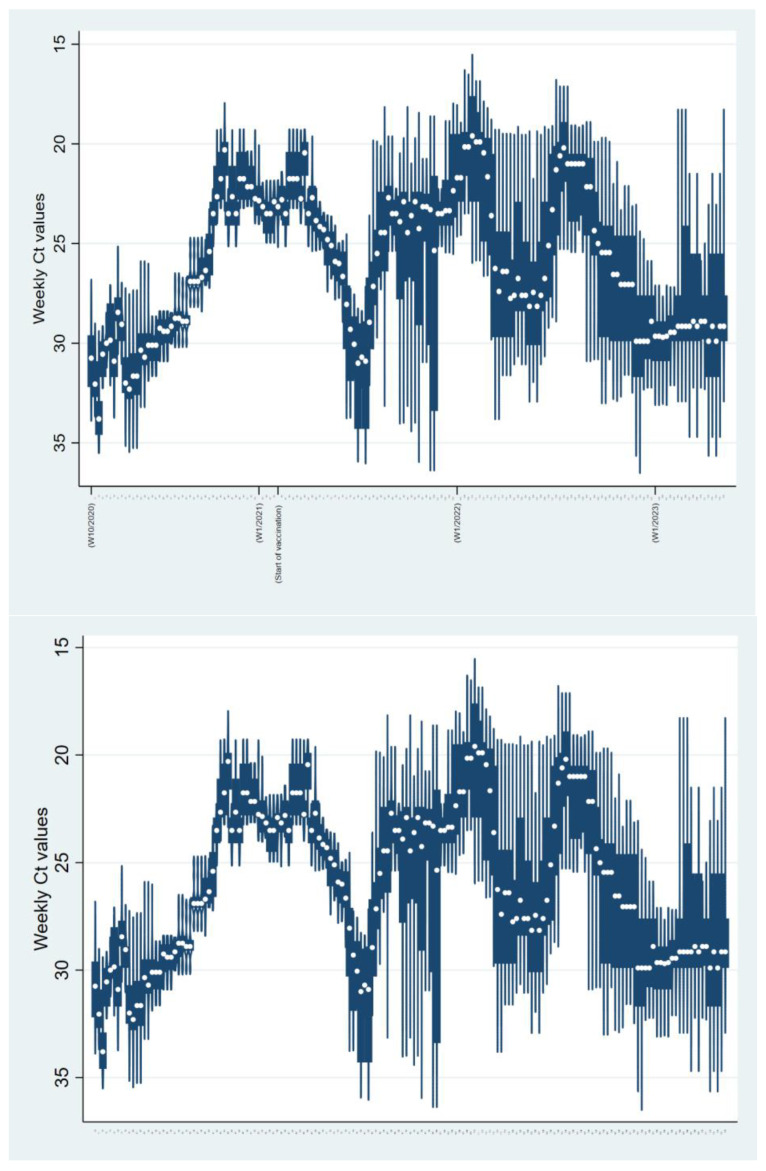
The distribution of cycle threshold (Ct) values per week (period April 2020–April 2023) is represented as a violinplot. The distribution of Ct values changed over time. The proportion of samples with low Ct increased with the proportion of positive tests and decreased when infection waned, indicating the end of the epidemic in 2023. The shown increment size is every ten weeks covered by the study, starting with the first and ending with the last week. The number of the week and the corresponding year to which the displayed data refer are given in parentheses.

**Figure 4:**
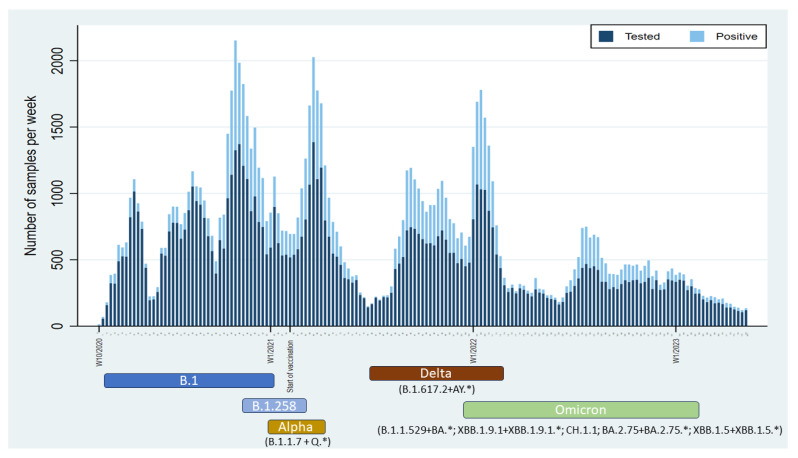
Weekly distribution of tested individuals and positive cases from April 2020 to April 2023. The x–axis denotes the beginning of each epidemic year and the initiation of vaccination. The bars represent the total number of tests conducted (labelled “Tested”) and the number of individuals testing positive for SARS-CoV-2 infection (labelled “Positive”). Colored boxes below the x–axis denote the predominant SARS-CoV-2 variants identified in Bosnia and Herzegovina, based on data from the GISAID database.

**Figure 5:**
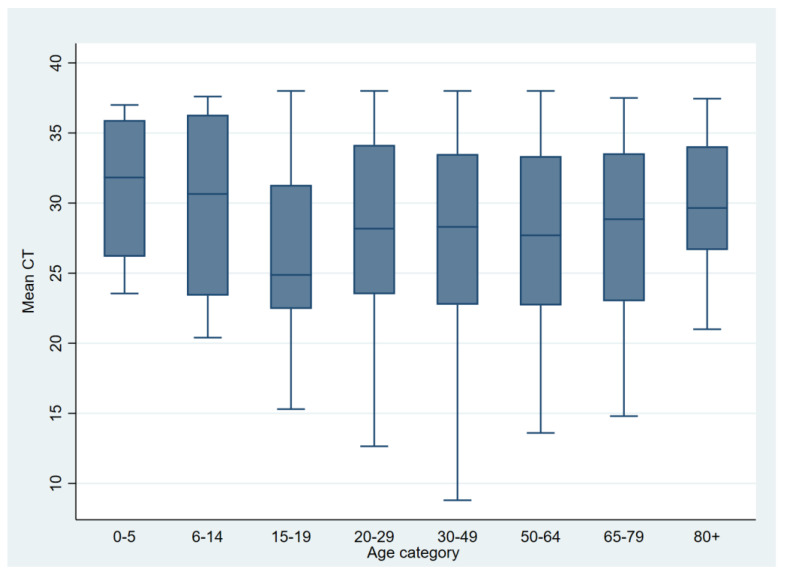
The overall median Ct values are represented per age group. The highest median Ct values had the youngest age groups (newborns/toddlers and preschool children—0–5 years) and the oldest age groups (seniors—80+ years). While trends in median Ct values are evident, statistical analysis revealed no significant differences between age groups.

The median weekly Ct values fluctuated between 19.9 and 33.2 throughout the epidemic. Lower median Ct values were inversely related to the number of positive cases, i.e., those Ct values corresponded to an increase in the population’s SARS-CoV-2 load. Furthermore, lower median Ct values were associated with the sharp increase in positive samples and, consequently, positive cases approximately 3–5 weeks thereafter (Figure 3), while higher median weekly Ct values (corresponding to a reduction in SARS-CoV-2 burden in the population) indicated the beginning of a receding recent outbreak/wave (Figure 2 and Figure 3). The Mann–Kendall test revealed a statistically significant monotonic trend in the data (*p* < 0.001), indicating a consistent directional change over the study period. This trend stopped at the end of 2022 and was not observed in 2023 because the increase of positively tested samples was not strictly followed by a decrease in median weekly Ct values (Figure 2). Individual Ct values ranged from 12.5 to 38 and were unequally distributed. The distribution of Ct values altered over time (Figure 3). The proportion of samples with low Ct increased with the proportion of positive tests, but this proportion gradually decreased as the outbreak/wave or infection weakened. An increase in the proportion of positive samples with low Ct values was associated with an introduction and subsequent circulation of SARS-CoV-2 variants of interest such as B.1.617.2 + AY.* (Delta) and B.1.1.529 + BA.* (Omicron), as can be seen with the shift between w53—2021 and w06—2023 (Figure 4). Overall, the annual weekly Ct values steadily decreased from approximately 28.7 to 23.6 during the epidemic waves, but after the dominance of the Omicron variant/subvariants (XBB.1.9.1 + XBB.1.9.1.*; CH.1.1; BA.2.75 + BA.2.75.*; XBB.1.5 + XBB.1.5.*) was established, the weekly median Ct values gradually increased to values similar to those at the beginning of the epidemic (Figure 3). The overall median Ct values per age group are depicted in Figure 5. The youngest age groups, including newborns/toddlers and preschool children (0–5 years), exhibited the highest median Ct values. Conversely, the secondary/high school children, comprising individuals aged 15 to 19 years, exhibited the lowest median Ct values. Nevertheless, our analysis did not yield statistically significant differences among any of the age groups (*p* = 0.31). Our analysis also encompassed an examination of Ct values obtained from both vaccinated and unvaccinated populations, spanning various age categories. Likewise, statistical analysis revealed no significant differences in Ct values between vaccinated and unvaccinated individuals across all age groups included in the study, indicating a consistent pattern across the different age groups.

## 4. Discussion

The inability to predict fluctuations in COVID-19 cases during the pandemic [16] has significantly impacted public health professionals’ ability to manage the situation. Shortly after the pandemic officially started in Bosnia and Herzegovina (the first positive case was in March 2020), health professionals, together with competent state authorities, declared a state of natural disaster [17]. Subsequently, in May 2020, restriction measures were introduced and all routine healthcare procedures were cancelled in anticipation of a rise in cases that did not occur. Furthermore, after the restrictions were relaxed, some clinical centers followed the valid precautionary measures but did not postpone routine practices any longer and were later overrun by an unanticipated rise in COVID-19 cases.

In this study, we analyzed the Ct value with the intention of determining the correlation between the Ct value and the SARS-CoV-2 incidence and course of the epidemic. The proportion of individuals who tested positive showed a steady increase which was not statistically correlated to gender or age group. Ct values showed a bimodal distribution previously described in viral infections [18] and observed in cases of SARS-CoV-2 [19]. Samples with low Ct values indicated that viral load was elevated during the acute phase of the disease, infectious stage, and high infectivity stage, while higher Ct values were typical of early infection or convalescence [8,20]. The overall analysis and distribution of Ct values revealed that the average weekly Ct values fluctuated during the outbreak and that the weekly mean Ct values gradually decreased (Figure 2 and Figure 3) and the peak increased overwhelmingly around lower Ct values (Figure 4). This supports recent virological surveys demonstrating that SARS-CoV-2 community Ct values are a useful early warning epidemiological indicator of changes in transmission [9,10]. Hay et al. [6] found that the distribution of Ct values at the population level strongly correlated with estimates of effective reproductive numbers of infection. This suggests that the epidemic trajectory or infection growth rate in the population could be inferred by analyzing the distribution of Ct values. However, they expressed their concerns that changes in test availability, testing methods, strategies, and phenotypical features of the virus could alter the distribution of Ct, potentially reducing the inferential power of this distribution. Similarly, changing SARS-CoV-2 mitigation strategies (easing the precautionary measures and governmental restrictions), as well as the emergence of new SARS-CoV-2 variants (changing phenotypical features of the virus), have been observed in Bosnia and Herzegovina in the past three pandemic years. Therefore, our results should be interpreted with caution and warrant further studies that will include data from other diagnostic laboratories in Bosnia and Herzegovina.

Dominant variants of concern (Alpha, Delta, and Omicron) have differed in their ability to evade immunization, viral loads, and incubation and shedding times [21]. Infections with the Delta variant resulted in even greater increases in RNA viral load compared to infections with the Alpha variant. A higher viral load associated with the Delta variant infection could mean that changes in Ct values might potentially overestimate the future number of cases. On the other hand, patients infected with Omicron BA.1 had lower viral loads than those infected with the Delta variant strain, which could potentially bias the estimation of the epidemic course in the near future. Compared to BA.1, the Omicron variant BA.2 infections cause higher levels of RNA virus loads, as reviewed in Puhach et al. [22]. These findings are in line with the median Ct value changes by the end of 2022, as the weekly median Ct values steadily rise. With the emergence of the BA.2 subvariant and concurrent changes in state policy that reduced population testing and potentially increased the number of tests in symptomatic patients, the median Ct values gradually declined, starting in w06 of 2022, to the levels recorded at the beginning of the pandemic. Such changes in state policy could limit the assessment of observed Ct values and potentially mean that the new epidemic wave may not be predicted. Alternatively, in this situation (the emergence and subsequent dominance of the BA.2 subvariant), a gradual but constant increase in Ct values, along with a decrease in weekly positivity rates, could indicate a decline in the epidemic in Bosnia and Herzegovina. The phenomenon that a rising epidemic necessarily has a high proportion of recently infected individuals with high viral loads, while a declining epidemic has more individuals with older infections and therefore lower viral loads, has previously been described [6]. However, taking into account all confounders, the general trends of the increased number of COVID-19 cases shortly after the drop in the weekly average Ct values are still largely successfully captured.

Our study investigated the distribution of Ct values in different age categories with the aim of identifying which population categories may contribute the most to the spread of SARS-CoV-2 infection. Our data revealed that the youngest age categories—newborns/toddlers and preschool children (0–5 years), group 2: elementary school children (6–14 years), and the oldest age category of the population (seniors/old population (80+ years))—had the highest Ct values. This is consistent with the results of previous studies which reported that children are less susceptible to SARS-CoV-2 infection [11], are usually asymptomatic compared to adults [23], and are rarely the index case in the transmission of the virus in the household [24]. Accordingly, the number of tested cases of the youngest and oldest age categories in our study was proportionally the smallest compared to other age categories. However, unlike children who were found to be less susceptible to SARS-CoV-2 infection [11], caution is needed in the interpretation of relatively high Ct values in the oldest age category of the tested population. Namely, the vast majority of deaths associated with SARS-CoV-2 infection were recorded in the oldest categories of the population [14]. Since seniors are an inactive population, the chain of transmission originates from people with whom they come into contact. Considering they are inactive, the testing time after showing the first symptoms of SARS-CoV-2 infection could be prolonged and, consequently, the Ct values established in these samples could be somewhat higher.

On the other hand, the largest number of prolonged positive cases was recorded in the adults/active working population (30–49 years) and retired population (65–79 years). Prolonged positive cases were considered to be people who had a positive result for SARS-CoV-2 for more than 20 days after an initial PCR-positive test (Figure 1). According to published data, prolonged persistence of viral RNA in samples is found regardless of spectrum of illness (ranging from asymptomatic to severely ill cases), indicating, but not exclusively, either high viral load and delayed viral clearance, or an inappropriate immune response unable to promote RNA virus clearance [25]. The finding of prolonged positive cases in the active working population and retired population could partially explain the mode of transmission of the SARS-CoV-2 virus in Bosnia and Herzegovina. Considering the fact that the population had no obligation to retest after the mandatory isolation and quarantine measures were implemented, the prolonged positive cases could have been the significant mode of transmission of the virus in the population of Bosnia and Herzegovina. It is important to consider that variations in RNA extraction and swabbing methods could influence these results. The observed differences in Ct values might reflect genuine epidemiological trends, but they could also be partially attributable to variability in the sample collection. However, since we did not test for virus viability, and considering that prolonged viral RNA detection does not necessarily indicate prolonged infectiousness, further research should incorporate standardized RNA normalization methods to better understand the relationship between age, Ct values, and disease dynamics.

There are several limitations observed in our study. While our study provides valuable insights into the trends of SARS-CoV-2 Ct values, it is important to acknowledge the potential confounding effects introduced by the aggregation of data from multiple laboratories and the use of different PCR kits. These variables, along with differences in sample collection methods, may contribute to variability in Ct values and could influence the interpretation of our findings. Considering this, we have calibrated PCR assays with known reference samples and implemented inter-laboratory comparisons to harmonize results. Despite efforts to mitigate these confounding factors, including the analysis of a substantial number of samples representing approximately one tenth of all COVID-19 tests conducted in Bosnia and Herzegovina which reduces the potential confounding and selection biases, it is imperative to recognize the inherent limitations in generalizing our results. However, the use of different PCR tests is a practice that cannot be avoided anywhere in the world and represents a limitation in studies of this type. Nevertheless, we have observed the same and consistent COVID-19 epidemiological trends, regardless of the laboratory and RT-qPCR tests used in the whole state territory. 

One of the potential limitations of our study is the variability in Ct values due to differences in RNA concentrations, which can arise from variations in swab collection methods and personnel. This variability is compounded by RT-qPCR variability and sample factors such as collection, storage, and sample type [12]. However, all samples were collected by medical staff, who were trained using standardized protocols to ensure consistent sample collection and handling. Internal controls in each reaction allowed us to monitor and exclude any samples with suboptimal extraction or amplification, enhancing the reliability of our Ct values. Furthermore, by using a single extraction method per batch, we minimized variability introduced in different extraction methods. Importantly, the two laboratories whose data were processed in our study are accredited according to ISO standards 17025 (general requirements for the qualification of testing and calibration laboratories) and 15189 (medical laboratories—requirements for quality and qualification). These accreditations mandate adherence to rigorous standards that foresee potential inconsistencies, thereby minimizing their impact through routine internal and external laboratory review and verification. Hence, by following the requirements of these ISO standards, the influence of the aforementioned factors is minimized. Despite these measures, we acknowledge that some variability may still exist. Future studies should continue to refine standardization methods for sample collection and processing to further reduce this variability. Another limitation of our study is that we did not account for the days post-symptom onset (DPSO) when analyzing Ct values. The DPSO could substantially influence Ct values as viral load dynamics vary throughout infection. Hence, our study might not fully capture these variations, potentially affecting the interpretation of Ct values concerning epidemiological trends. However, it is important to note that our primary focus is on population-level trends rather than on individual-level variations. While DPSO could eventually mask some epidemiological trends, the fluctuations in Ct values at the population level can still serve as a useful additional epidemiological tool. The consistency of our results across large sample sizes and multiple testing sites helps mitigate potential variability introduced by not accounting for DPSO.

The chosen Ct value threshold of 38, as recommended by the diagnostic kit manufacturers, ensured high sensitivity by detecting lower viral loads, which is crucial for identifying early or asymptomatic infections. However, this higher threshold might reduce specificity, potentially leading to false-positive results due to the detection of non-viable RNA remnants.

On the other hand, this study demonstrated that analyzing Ct values could be helpful in the study of SARS-CoV-2 infection and the COVID-19 pandemic. Prompt measurement of Ct values can identify which specific or high-risk population subgroups are more likely to be affected by the virus. The SARS-CoV-2 load in the upper respiratory tract is considered to be a predisposing factor in the transmission risk [25]. Also, the viral load is inversely related to Ct values, i.e., lower Ct values may indicate a higher viral load in the individual confirmed to be positive for SAR-CoV-2. Consequently, the gradual or sharp decrease in weekly median Ct values might indicate an upcoming increase in the number of positive individuals, i.e., the outbreak or even an epidemic wave. Therefore, this study recommends that public health authorities should consider collecting these epidemiologic data from major diagnostic laboratories as an important part of epidemiological surveillance. Observing the changes in weekly Ct values might serve as an early indicator for detecting the epidemic growth and an additional tool for timely reaction and implementation of all necessary precautionary measures to prevent the introduction and, subsequently, high positivity rates and consequentially increased mortality in high-risk populations, not only in the COVID-19 pandemic but also in future pandemics that could happen. Additionally, the changes in Ct values might be the first indicator for the possible changes in phenotypic characteristics of the circulating variants of SARS-CoV-2. Our study warrants the conducting of further studies aimed at determining if Ct values vary according to other important COVID-19 risk factors. The results of our study indicate the possibility of a simple assessment of the relative burden of future cases of COVID-19 in the public health system. Public health decision-makers might use aggregated Ct values to estimate the timing of the next epidemic wave and more effectively plan and implement appropriate outbreak measures in surveillance settings, such as in Bosnia and Herzegovina. Additionally, analyzing aggregated Ct values could offer better guidance for resource allocation. Ct values of SARS-CoV-2, obtained through routine testing, can provide further insights for predicting disease outbreaks and making more informed decisions about resource use. Future research could benefit from integrating quantitative viral load measurements for a more comprehensive understanding of viral infectivity. Exploring the relationship between Ct values, viral load, and culturable viruses would enhance the interpretation of infectiousness and improve public health strategies.

## Data Availability

Dataset available on request from the authors.

## Supplementary Materials

The following supporting information can be downloaded at: https://www.mdpi.com/article/10.3390/microorganisms12081585/s1, Table S1: the list of diagnostic kits used, with accompanying information about the manufacturer, target genes, internal control, and appropriate detection fluorophore channels.
