# Supplementary material for: Trends in SARS-CoV-2 Cycle Threshold Values in Bosnia and Herzegovina—A Retrospective Study
Source: Microorganisms. 2024 Aug 4;12(8):1585. doi: 10.3390/microorganisms12081585 (PMC11356242; doi:10.3390/microorganisms12081585)
Supplement: Supplementary file 1 [file microorganisms-12-01585-s001.zip › microorganisms-3117070-supplementary.pdf]

Supplementary Table S1. The list of used diagnostic kits with accompanying information about the manufacturer, target genes, internal control and appropriate detection fluorophore channels.

**Diagnostic CE-IVD kit**	**Manufacturer**	**Target gene(s)**	**Detection channel**	**Internal control**	**Detection channel**
Fosun COVID-19 RT-PCR Detection Kit	Fosun Diagnostics, Shanghai, China	ORF1ab gene<br>N gene<br>E gene	FAM<br>JOE<br>ROX	Internal reference	Cy5
Multiple Real-Time PCR Kit for Detection of 2019-nCoV	X-ABT Beijing Applied Biological Technologies, Beijing, China	ORF1ab gene<br>N gene<br>E gene	FAM<br>VIC<br>ROX	RNase P	Cy5
FTD SARS-CoV-2 Assay	Siemens Healthineers, Erlangen, Germany	ORF1ab gene<br>N gene	FAM*	Equine Arteritis Virus	Cy5
LabGun™ COVID-19 RT-PCR Kit	LabGenomics, South Korea	RdRp gene<br>E gene	FAM<br>Cy5	MS2 RNA internal control	VIC/HEX
PROmate® COVID-19 2G Kit	Primerdesign™ Ltd, Dayton, USA	ORF1ab gene<br>Nsp16 gene	FAM<br>Cy5	RNA template	HEX
GeneFinder™ COVID-19 Plus RealAmp Kit	Osang Healthcare, South Korea	RdRp gene<br>E gene<br>N gene	FAM<br>Texas Red<br>JOE/VIC	RNase P	Cy5
Lilly SARS- CoV-2 Assay	Eli Lilly and Company, Indianapolis, USA	N1 gene<br>N2 gene	FAM*	RNase P	Cy5
Bosphore Novel Coronavirus (2019-nCoV) Detection Kit v2	Anatolia Genworks, Turkey	ORF1ab gene<br>E gene	FAM<br>HEX	Internal Control	Cy5

*- FTD SARS-CoV-2 Assay and Lilly SARS- CoV-2 Assay are single-well, dual target assays
